# Lingual Kinematics in Dysarthric and Nondysarthric Speakers with Parkinson's Disease

**DOI:** 10.4061/2011/352838

**Published:** 2011-10-09

**Authors:** Min Ney Wong, Bruce E. Murdoch, Brooke-Mai Whelan

**Affiliations:** Centre for Neurogenic Communication Disorders Research, School of Health and Rehabilitation Sciences, The University of Queensland, St Lucia, QLD, 4072, Australia

## Abstract

Articulatory dysfunction is recognised as a major contributor to the speech disturbances seen in Parkinson's disease (PD). The present study aimed to compare lingual kinematics during consonant production within a sentence in eight dysarthric (DPD) and seven nondysarthric (NDPD) speakers with PD with those of eleven nonneurologically impaired normal participants. The tongue tip and tongue back movements of the participants during sentence production were recorded using electromagnetic articulography (EMA). Results showed that both the DPD and NDPD had deviant articulatory movement during consonant production that resulted in longer duration of consonant production. When compared with the NDPD group, the DPD group primarily exhibited increased range of lingual movement and compatible duration of production with an accompanying increase in maximum velocity, maximum acceleration, and maximum deceleration. These findings are contrary to proposed theories that suggest articulatory imprecision in dysarthric speakers with PD is the outcome of reduced range of articulatory movement.

## 1. Introduction

Idiopathic Parkinson's disease (PD) is a movement disorder typically presenting with motor symptoms such as slowness of movement, muscle rigidity, tremor, and postural instability. Dysarthria, a product of a movement disorder involving the muscles of the speech production mechanism [1], has been frequently identified in individuals with PD, with the majority of the studies reporting an increase of dysarthria prevalence in both numbers and severity with the progress of the disease [2, 3]. Previous studies reported that imprecision of consonant production is the most common articulatory impairment in individuals with PD who suffered a hypokinetic dysarthria [4–9]. Logemann and Fisher [10] reported that stops, affricates, and fricatives were often distorted in their participants with PD. Darley et al. [5–7] hypothesised that the imprecision of consonant production is the result of limited range of movement, muscle rigidity, slowness of movement, and reduced force of movement of the articulators. Thus, direct investigation of articulatory movement, especially tongue movement, during speech production would contribute to the understanding of the nature of articulatory deficits in individuals with PD. 

To date, a limited number of studies have used electromagnetic articulography (EMA) to investigate the articulatory function in individuals with PD. Ackermann et al. [11] conducted a case study in an akinetic-rigid dysarthric woman with PD using EMA. The authors investigated labial and lingual function of the participant during diadochokinesis/rapid syllable repetition tasks and reported occurrence of speech freezing during the production of /ta/ repetitions accompanied by increased repetition rates and reduced amplitude of tongue movement. The speech freezing was characterised by the production of a sustained /a/ instead of the /ta/ repetitions. These kinematic findings supported the hypothesis of articulatory undershoot (i.e., reduction in movement amplitude of the articulators) in individuals with PD [12]. Wong et al. [13] investigated the lingual function during consonant production within a sentence in a group of nondysarthric speakers with PD. The study reported that the nondysarthric speakers with PD had a similar range of tongue movement as controls. Furthermore, the nondysarthric PD group showed a reduction in movement parameter values (i.e., velocity, acceleration, and deceleration) in the approach phase of consonant production which was compensated by an increase in the duration of tongue movement. In the release phase of consonant production, comparable movement parameter values were reported between the nondysarthric PD group and the control group. The authors hypothesised that reduction in the range of tongue movement may be the main feature that distinguishes dysarthric speakers with PD from nondysarthric speakers with PD. 

In a further study, Wong et al. [14] explored the lingual kinematics during consonant production within a sentence in a group of dysarthric speakers with PD. The study documented that their dysarthric speakers with PD, when compared to controls, had a comparable range of lingual movement during alveolar consonant production but an increased range of lingual movement during velar consonant production. In contrast to their findings in nondysarthric speakers with PD [13], Wong et al. [14] reported that the dysarthric PD group, when compared to controls, had increased movement parameter values (i.e., velocity, acceleration, and deceleration) predominantly in the release phase of consonant production. Despite the increase in movement parameter values, when compared to controls, the dysarthric PD group had comparable duration of lingual movement during velar consonant production and increased duration of lingual movement during alveolar consonant production. Based on the findings on dysarthric speakers with PD [14], the authors suggested that the imprecision of articulatory production may be due to the increased range of lingual movement in the release phase of consonant production. 

The current study is a followup to the earlier reports of Wong et al. [13, 14]. These earlier reports examined the lingual kinematics in nondysarthric speakers with PD and dysarthric speakers with PD separately, and no between groups comparison had been carried out. In order to determine the factors that may contribute to dysarthria in PD, a comparison between the nondysarthric speakers with PD and dysarthric speakers with PD is essential. The focus of the present study, therefore, is to compare lingual kinematics in dysarthric speakers with PD, nondysarthric speakers with PD, and a group of normal (non-neurologically impaired) participants using EMA. However, there were some methodological differences between Wong et al. [13] and Wong et al. [14] that necessitated a reanalysis of data. The data on duration and distance of tongue movements in Wong et al. [13] were derived on the basis of analysis of an entire sentence production while the data on duration and distance of tongue movements in Wong et al. [14] were analysed based on each separate gesture (i.e., approach and release phases of the target consonant). Hence, the present study reanalysed the data on duration and distance of tongue movement from Wong et al. [13] based on each separate gesture to match with the data from Wong et al. [14]. It was hypothesised that the dysarthric speakers with PD would show impairment in lingual kinematics measures including velocity, acceleration, deceleration, distance, and duration, compared to nondysarthric speakers and normal participants. 

## 2. Methods

### 2.1. Participants

The present study involved three groups of participants, a dysarthric PD group (DPD), a nondysarthric PD group (NDPD), and a normal group. The classification of dysarthric or nondysarthric PD group was made based on the findings of a perceptual analysis. Perceptual speech samples were obtained during the reading of the Grandfather Passage [7] and were recorded using a SONY Portable Minidisk Recorder MZ-R700. Two experienced speech-language pathologists, who were blinded to the history and the neurological status of each participant, were employed to evaluate the speech samples independently. The perceptual rating scales utilised were devised by FitzGerald et al. [15]. The rating scales included 32 speech dimensions covering the five aspects of speech production, that is, prosody, respiration, phonation, resonance, and articulation. In cases of discrepancies, the two experienced speech-language pathologists discussed among themselves and produced a single consensus rating to be used in all further analysis. During the rating of speech samples, the two experienced speech-language pathologists were also required to determine the presence and severity of any associated dysarthria. A participant was judged to be nondysarthric if the participant showed not more than a just noticeable level of deviation in a maximum of one of the 32 speech dimensions rated [13]. 

The DPD group comprised eight participants (six males and two females) with a mean age of 66.66 years (SD = 6.61) and an age range of 56 to 78 years. All participants in the DPD group were perceptually judged to present with a mild hypokinetic dysarthria. The NDPD group consisted of seven participants (two males and five females), with a mean age of 60.03 years (SD = 6.53) and an age range of 49 to 68 years. All participants with PD were native English speakers and had been diagnosed with idiopathic PD by a neurologist. Individuals who had a history of a neurological condition other than PD, neurosurgical intervention including deep brain stimulation, speech disorders, speech therapy, or oromaxillofacial surgery involving the tongue and/or lip were excluded from the study. All participants with PD also had adequate hearing for the purpose of this study. The biographical details of the dysarthric and nondysarthric speakers with PD are shown in Table 1. (Note: all participants included in the DPD and NDPD groups had previously been included in the studies reported by Wong et al. [14] and Wong et al. [13] resp.). 

A group of 11 non-neurologically impaired, native English speaking individuals matched for age and sex served as the normal group. All participants included in the normal group had previously been included in the studies reported by Wong et al. [13] and/or Wong et al. [14]. The mean age of the normal group was 64.52 years (SD = 8.31) with an age range of 49 to 78 years. All participants in the normal group were free from a history of neurological/speech disorder, respiratory disease, substance abuse, or oromaxillofacial surgery. The speech of all participants in the normal group was also perceptually rated in the same manner as the PD groups with all participants in the normal group judged to have within normal limit performance for all dimensions rated. The study protocol was approved by the respective ethical review committee at The University of Queensland, QLD, Australia and all participants provided written informed consent. 

### 2.2. Procedures

The movement of the tongue tip and tongue back during speech production in each participant was investigated using the electromagnetic articulograph (EMA) AG-200 system (Carstens Medizinelektronik GmbH, Germany). The present study employed the methodologies outlined in Wong et al. [14]. Participants with PD were assessed in an “on” medication phase, approximately one hour after medication. 

The EMA system has three electromagnetic transmitter coils fixed in the midsagittal plane of a light-weight plastic helmet suspended around the participant's head. Five miniature receiver coils (approximately 2 × 2 × 3 mm in size) are affixed to the midline of the participant's articulators, that is, bridge of nose, maxilla above the upper central incisors, tongue tip (1 cm from tongue tip), tongue back (4 cm from tongue tip), and the jaw (under the chin). When the three transmitter coils generate alternating magnetic fields at different frequencies (range 10–20 kHz), alternating signals are induced in the receiver coils. The magnitude of these signals is used to calculate the distance between a single receiver coil and a transmitter coil and to determine the *x*-*y* positions of the receiver coil. In the present study, the sampling rate of the position of each of the receiver coils was set at 200 Hz. A lapel microphone was used to record the speech acoustic signals at a sampling rate of 16 kHz. 

The participants were required to use their normal speaking voice to read aloud the alveolar sentence *Tess told Dan to stay fit *and the velar sentence *Karl got a croaking frog* randomly five times each. A sentence repetition task was adopted as it approximates more closely the condition of natural speech as compared to syllable repetition and word production tasks. Due to the difficulties in keeping the receiver coil in situ for the duration of the EMA assessment, velar data were not recorded for one of the participants in the normal group. For each sentence, only three productions free of error and dysfluencies were selected from each participant for analysis. All alveolar consonants from the alveolar sentence and all velar consonants from the velar sentence were included in the analysis. The data was then averaged across consonants in each sentence. 

In preparation for data analysis, the Tailor program 1.3 (Carstens Medizinelektronik GmbH, Germany) was used to filter the data and correct for head movement in relation to the helmet. Firstly, the two reference channels were filtered using Filter 40 (cutoff = 8 Hz), and the three data channels were filtered using Filter 160 (cutoff = 32 Hz). Dynamic correction was then conducted to correct for any head movement in relation to the helmet. Finally, the movement data were rotated within the *x-y *plane to ensure that the occlusional plane was parallel to the *x*-axis. This step helped to ensure that the orientation of kinematic data was consistent between participants. 

**Figure 1:**
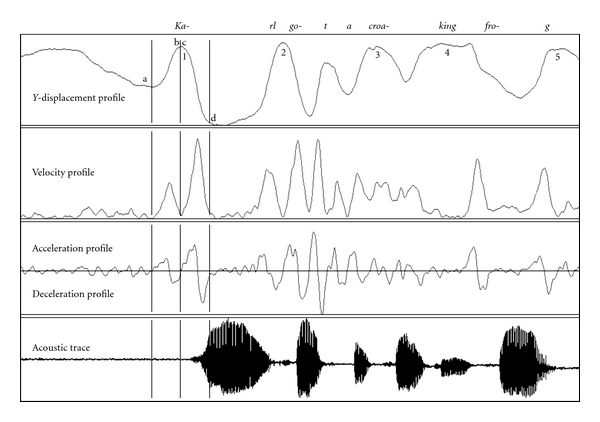
Example of tongue back movement in a participant with PD during the production of *Karl got a croaking frog.* 1–5: peaks for analysis, a: start of approach, b: end of approach, c: start of release, d: end of release. Duration of approach phase [(b-a) × 5], and release phase [(d-c) × 5], given a sampling rate of 200 Hz.

The data were then loaded into the Emalyse program 3.91 (Carstens Medizinelektronik GmbH, Germany) for data analysis. The *y*  displacement, velocity, and acceleration profiles were used to determine maximum velocity (mm/s), maximum acceleration/deceleration (m/s^2^), and segmentation of the approach phase (tongue movement up to the palate) and the release phase (tongue movement away from the palate) during consonant production across the sentence (see Figure 1). The duration (ms) of consonant production and distance (mm) travelled were also calculated. A custom-written script in Matlab (The Mathworks, Inc., MA, USA) was used to calculate the distance travelled in each phase during consonant production. The acoustic trace helped to isolate the individual consonants for analysis.

### 2.3. Statistical Analyses

The data were first screened for outliers prior to statistical analysis. For the purpose of this study, a *z*-value of 3.29 constituted an outlier [16] and was replaced with the group mean. Subsequently, a nonparametric technique, the Kruskal-Wallis test, was applied for comparison between the DPD, the NDPD, and the normal groups due to unequal sample sizes. Post hoc testing utilised the Mann-Whitney *U* test to compare articulatory kinematics between the groups. The alpha level of 0.05 was adopted for significance. 

## 3. Results

**Figure 2:**
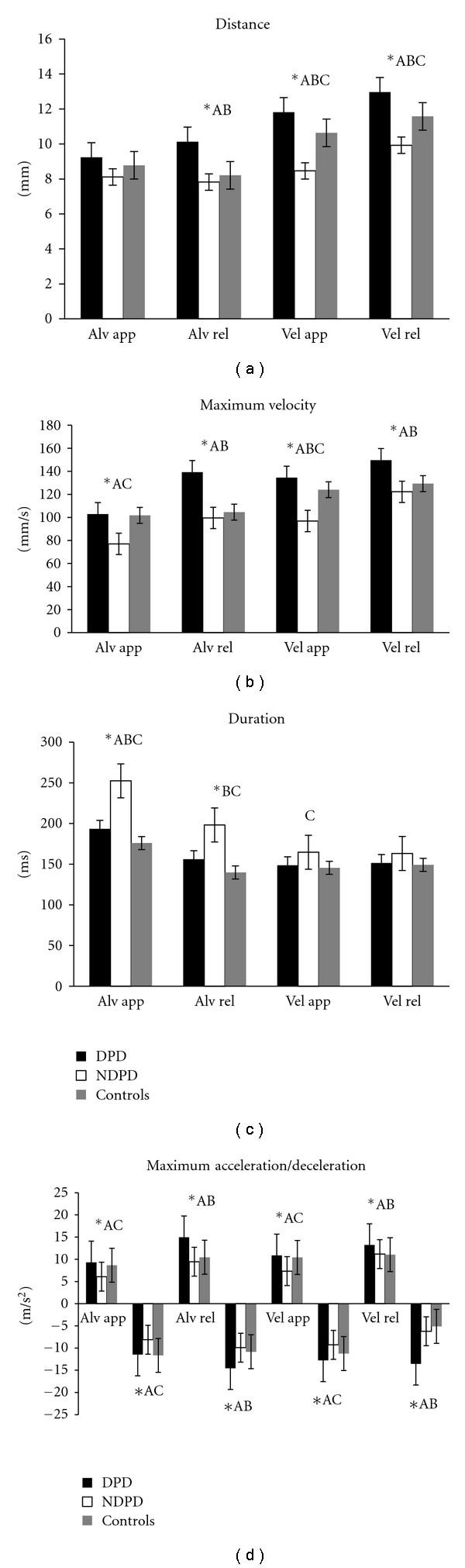
Mean (and standard error) kinematic parameter values recorded for the DPD, NDPD and normal groups. DPD: dysarthric speakers with Parkinson's disease, NDPD: nondysarthric speakers with Parkinson's disease, Alv: alveolar sentence, Vel: velar sentence, app: approach phase, and rel: release phase, Significant differences between group comparison: ^*∗*^= DPD versus NDPD versus controls, A: DPD versus NDPD, B: DPD versus controls, C: NDPD versus controls.

The mean and standard error of kinematic parameters for the DPD, the NDPD, and the normal groups together with the results of the Kruskal-Wallis test and the Mann-Whitney *U* test are displayed in Figure 2.

### 3.1. DPD versus NDPD versus Normal

The results of the Kruskal-Wallis test showed statistically significant differences across the DPD, the NDPD, and the normal groups in maximum velocity, maximum acceleration, and maximum deceleration of tongue movement in both the approach (*P* ≤ 0.001) and release (*P* < 0.05) phases of alveolar and velar consonant production. Distance of tongue movement was significantly different across groups in the release (*P* < 0.01) phase of alveolar consonant production and in both the approach (*P* < 0.001) and the release (*P* < 0.001) phases of velar consonant production. The duration of production was only significantly different across groups during alveolar consonant production in both the approach (*P* < 0.001) and the release (*P* < 0.001) phases. The three groups of participants had comparable duration of consonant production in both the approach and the release phases of velar consonant production. Post hoc analysis using the Mann-Whitney *U* test revealed the following results.

### 3.2. DPD versus NDPD

When compared to the NDPD group, the DPD group had significantly increased distance of tongue movement in the release (*P* < 0.01) phase of alveolar consonant production and in both the approach (*P* < 0.001) and the release (*P* < 0.001) phases of velar consonant production. The DPD group also showed significantly increased maximum velocity, maximum acceleration, and maximum deceleration of tongue movement in both the approach (*P* < 0.001) and the release (*P* < 0.05) phases of alveolar and velar consonant production. The duration of consonant production was observed to be comparable between the DPD group and the NDPD group in the release phase of alveolar consonant production and in the approach and the release phases of velar consonant production. The DPD group had shorter duration of consonant production in the approach (*P* < 0.01) phase of alveolar consonant production.

### 3.3. DPD versus Normal

The DPD group, when compared to the normal group, had significantly increased maximum velocity, maximum acceleration, and maximum deceleration of tongue movement, predominantly in the release phase of both alveolar (*P* ≤ 0.001) and velar (*P* < 0.05) consonant production. The distance of tongue movement was significantly increased in the DPD group, evidenced in the release (*P* < 0.01) phase of alveolar consonant production and in both the approach (*P* < 0.05) and the release (*P* < 0.05) phases of velar consonant production. The DPD group also had significantly longer duration of consonant production than normal group in both the approach (*P* < 0.01) and the release (*P* < 0.001) phases of alveolar consonant production. However, comparable performance was observed between the DPD and the normal groups during the velar consonant production in both the approach and the release phases. 

### 3.4. NDPD versus Normal

The distance of tongue movement in the NDPD group, when compared to the normal group, was significantly reduced in the approach (*P* < 0.001) and the release (*P* < 0.05) phases of velar consonant production while comparable performance was observed in the approach and the release phases of alveolar consonant production. The maximum velocity, maximum acceleration, and maximum deceleration of tongue movement in the NDPD group were significantly reduced in the approach phase of both alveolar (*P* < 0.001) and velar (*P* < 0.05) consonant production, when compared to the normal group. Comparable maximum velocity, maximum acceleration, and maximum deceleration of tongue movement were observed in the release phase of both alveolar and velar consonant production. The duration of consonant production in the NDPD group was significantly longer than the normal group in both alveolar (*P* < 0.01) and velar (*P* < 0.05) consonant production excluding the release phase of velar consonant production. 

## 4. Discussion

The results of the present study documented the presence of deviant articulatory movements in both dysarthric and nondysarthric speakers with PD during the production of alveolar and velar consonants within a sentence. However, the DPD and the NDPD groups had different patterns of lingual movement deficits, when compared to the normal group; the DPD group had predominantly increased range of lingual movement while the NDPD group had comparable/reduced range of lingual movement; the DPD group had increased maximum velocity, maximum acceleration, and maximum deceleration predominantly in the release phase of consonant production while the NDPD group had reduced maximum velocity, maximum acceleration, and maximum deceleration predominantly in the approach phase of consonant production. Despite the different patterns observed, both the DPD and the NDPD groups had longer duration of consonant production than the normal group. When compared to the NDPD group, the DPD group had increased maximum velocity, maximum acceleration, and maximum deceleration in the production of both alveolar and velar consonants. While both the DPD and the NDPD groups had a similar range of lingual movement (in the approach phase of alveolar consonant production), the reduction in the duration of lingual movement in the DPD group appeared to be primarily the outcome of increased maximum velocity, maximum acceleration, and maximum deceleration. However, when the DPD group had increased the range of lingual movement (in the release phase of alveolar consonant production and both the approach and release phases of velar consonant production), the increased maximum velocity, maximum acceleration, and maximum deceleration in the DPD group seemed to offset the expected increase in the duration of lingual movement in the DPD group and render duration comparable between the groups. 

The findings of reduced range of movement, maximum velocity, maximum acceleration, and maximum deceleration in the NDPD group are consistent with previous perceptual, acoustic, and kinematic studies on speakers with PD that have reported reduced movement amplitude and/or movement velocity in dysarthric speakers with PD [5–7, 11, 12, 17, 18] and supports the hypothesis of articulatory undershoot in individuals with PD [12]. However, the articulatory undershoot hypothesis does not appear to explain the articulatory disturbance noted in the dysarthric speakers with PD included in the present study. Rather, the observed increased range of lingual movement and increased maximum velocity, maximum acceleration, and maximum deceleration in the DPD group provide a different perspective to the lingual movement deficits in individuals with PD. A study conducted by Forrest et al. [17] reported that their Parkinsonian speakers, when compared to normal geriatrics, had increased lower lip closing velocities expressed as a function of movement amplitude. It would appear that the alteration in lingual kinematics were strategies used by the DPD group to meet the demands of normal speaking condition. These strategies may represent an attempt by the participants with dysarthria to maintain speech rate involving an increase in the various speed parameters (i.e., maximum velocity, maximum acceleration, and maximum deceleration) with the concomitant increase in the range of movement representing a byproduct of the increased speed of movement of the tongue. However, this interpretation is at best speculative. This finding also suggests that the pathophysiology of lingual movement during speech production may be different from the pathophysiology of limb bradykinesia which is marked by slow and limited range of movement. Furthermore, these findings are contrary to the suggestion by Darley et al. [5–7] that the deviant speech dimensions observed in dysarthric speakers with PD are the product of a restricted range of articulatory movement. 

The increased speed parameters (i.e., maximum velocity, maximum acceleration, and maximum deceleration) in the DPD group during consonant production, as compared to the NDPD group and the normal group, may potentially be contributed to by various factors including drug-induced dyskinesia [19] and an abnormally large vocal tract in some participants [20]. The presence of drug-induced dyskinesia is unlikely as none of the participants with DPD included in the present study were reported by their neurologist to have dyskinesia. However, the effect of the sizes of vocal tract on speed parameters could not be ruled out with certainty as the present study did not incorporate volumetrics estimation of the vocal tract, including the oral cavity. The inclusion of a number of participants with abnormally large vocal tracts could be expected to lead to increased articulatory movement as observed in the present study. However, none of the individuals with PD included in the present study were noted to be abnormally large or small in body size, and; hence, such bias in our sample of individuals with PD is unlikely. Further, volumetric estimates of the oral cavity are difficult to perform with accuracy and for that reason have not been included in previous research into dysarthria in PD. It is recommend that such measures be considered for inclusion in future studies of articulatory function in PD and other neurological conditions to rule out possible effects of vocal tract size on articulatory movement. The impaired rate of speech in individuals with PD may also be related to sensorimotor deficits in the orofacial system and abnormal temporal, auditory, and perceptual processing of speech [21–26]. 

## 5. Limitations and Future Research

Although the present study employed a group analysis method in the examination of lingual kinematics in individuals with PD, previous studies have reported considerable interparticipant variations [18, 27]. Further detailed case discussion would shed light on our understanding of articulatory dynamics in individuals with PD as individual lingual kinematic characteristics may potentially have been masked in a group study. The present study has certain limitations with regards to the methodology employed. Firstly, all participants included in the DPD group were only mildly dysarthric, a factor which would have decreased the likelihood of finding differences in their lingual kinematics during consonant production, as compared to the NDPD group. The fact that differences in kinematics parameters were indentified between these two groups would appear to emphasise the advantage and importance of physiological assessments such as EMA, compared to perceptual assessments, in the determination of impaired physiological functioning of the articulators in conditions such as PD. Based on the findings of the present study, it is evident that the DPD and the NDPD groups each used different lingual kinematics strategies to achieve their current level of precision during consonant production. Overall, this has important clinical implications. Future studies should include participants with a wider severity of PD to further elucidate the effects of PD on articulatory function. Further, the analysis of alveolar and velar consonant production could be further expanded by examining the effect of vowel environment on lingual kinematics. It is also recommended that volumetric estimates of the oral cavity should be considered for inclusion in future studies of articulatory function in PD and other neurological conditions to rule out possible effects of vocal tract size on articulatory movement. The effect of speaking tasks and the use of stressed versus unstressed syllables could be examined in future studies as some previous research had suggested possible differences in articulatory movements [12, 28]. Dromey [29] and Goozée et al. [30] reported changes in articulatory movement in participants with PD under the influence of loudness. Given the increasing use of Lee Silverman Voice Treatment (LSVT) for speech rehabilitation in individuals with PD, future longitudinal kinematics studies examining the pre- and post-LSVT articulatory dynamics would facilitate our understanding of the articulatory changes as a result of the treatment. Future studies are also encouraged to incorporate a larger group of dysarthric speakers with different levels of severity. 

## 6. Conclusion

Impaired lingual kinematics was documented in both the dysarthric and nondysarthric speakers with PD as compared to normal participants, with different patterns of lingual kinematics observed. The NDPD group primarily exhibited a reduction in the range of lingual movement and reduced maximum velocity, maximum acceleration, and maximum deceleration accompanied by an increased duration of lingual movement. On the other hand, the DPD group primarily showed an increase in the range of lingual movement and increased maximum velocity, maximum acceleration, and maximum deceleration accompanied by an increased duration of lingual movement. The DPD group, when compared to the NDPD group, primarily had increased range of lingual movement and increased maximum velocity, maximum acceleration, and maximum deceleration accompanied by comparable duration of lingual movement. The findings from the current study are contrary to the proposed theories that suggest articulatory imprecision in dysarthric speakers with PD is the outcome of reduced range of articulatory movement. 

## Figures and Tables

**Table 1:** Biographical details of the dysarthric and nondysarthric speakers with Parkinson's disease.

Participant	Gender	Age (years)	Year after-PD onset	The Hoehn and Yahr stage	Medication
Dysarthric speakers with PD					
1	F	64	<1	1-2	Madopar
2	F	67	11	1	Sinemet, Cabaser
3	M	78	18	3	Sinemet, Comtan
4	M	71	11	2-3	Sinemet, Cabaser
5	M	56	9	2	Madopar, Comtan
6	M	69	6	1-2	Stalevo
7	M	67	3	1	Stalevo
8	M	61	6	2	Madopar, Sinemet, Symmetrel

Nondysarthric speakers with PD					
1	F	64	6	1–1.5	Sinemet, Cabaser
2	F	49	3	0-1	Sinemet, Cabaser
3	F	63	7	N/A	Sinemet, Cabaser
4	F	54	2	1–1.5	Sinemet, Cabaser
5	F	63	6	1–1.5	Sinemet, Comtan, Parlodel
6	M	68	6	3	Sinemet
7	M	59	10	1	Madopar, Comtan, Cabaser

Note: PD: Parkinson's disease; N/A: not available.

## References

[1] Kent RD, Netsell R, Bauer LL (1975). Cineradiographic assessment of articulatory mobility in the dysarthrias. *Journal of Speech and Hearing Disorders*.

[2] Johnson JA, Pring TR (1990). Speech therapy and Parkinson's disease: a review and further data. *British Journal of Disorders of Communication*.

[3] Scott S, Caird FI, Williams BO (1985). *Communication in Parkinson's Disease*.

[4] Chenery HJ, Murdoch BE, Ingram JCL (1988). Studies in Parkinson's disease. I. Perceptual speech analyses. * Australian Journal of Human Communication Disorders*.

[5] Darley FL, Aronson AE, Brown JR (1969). Clusters of deviant speech dimensions in the dysarthrias. *Journal of Speech and Hearing Research*.

[6] Darley FL, Aronson AE, Brown JR (1969). Differential diagnostic patterns of dysarthria. *Journal of Speech and Hearing Research*.

[7] Darley FL, Aronson AE, Brown JR (1975). *Motor Speech Disorders*.

[8] Logemann JA, Fisher HB, Boshes B, Blonsky ER (1978). Frequency and cooccurrence of vocal tract dysfunctions in the speech of a large sample of Parkinson patients. *Journal of Speech and Hearing Disorders*.

[9] Plowman-Prine EK, Okun MS, Sapienza CM (2009). Perceptual characteristics of Parkinsonian speech: a comparison of the pharmacological effects of levodopa across speech and non-speech motor systems. *NeuroRehabilitation*.

[10] Logemann JA, Fisher HB (1981). Vocal tract control in Parkinson's disease: phonetic feature analysis of misarticulations. *Journal of Speech and Hearing Disorders*.

[11] Ackermann H, Gröne BF, Hoch G, Schönle PW (1993). Speech freezing in Parkinson's disease: a kinematic analysis of orofacial movements by means of electromagnetic articulography. *Folia Phoniatrica*.

[12] Ackermann H, Ziegler W (1991). Articulatory deficits in Parkinsonian dysarthria: an acoustic analysis. *Journal of Neurology, Neurosurgery and Psychiatry*.

[13] Wong MN, Murdoch BE, Whelan B-M (2010). Tongue function in nondysarthric speakers with Parkinson's disease: an electromagnetic articulography investigation. *Journal of Medical Speech-Language Pathology*.

[14] Wong MN, Murdoch BE, Whelan B-M (2010). Kinematic analysis of lingual function in dysarthric speakers with Parkinson's disease: an electromagnetic articulograph study. *International Journal of Speech-Language Pathology*.

[15] FitzGerald FJ, Murdoch BE, Chenery HJ (1987). Multiple sclerosis: associated speech and language disorders. *Australian Journal of Human Communication Disorders*.

[16] Field A (2009). *Discovering Statistics Using SPSS*.

[17] Forrest K, Weismer G, Turner GS (1989). Kinematic, acoustic, and perceptual analyses of connected speech produced by Parkinsonian and normal geriatric adults. *Journal of the Acoustical Society of America*.

[18] McAuliffe MJ, Ward EC, Murdoch BE (2005). Articulatory function in hypokinetic dysarthria: an electropalatographic examination of two cases. *Journal of Medical Speech-Language Pathology*.

[19] de Letter M, Santens P, de Bodt M, Boon P, van Borsel J (2006). Levodopa-induced alterations in speech rate in advanced Parkinson's disease. *Acta Neurologica Belgica*.

[20] Kuehn DP, Moll KL (1976). A cineradiographic study of VC and CV articulatory velocities. *Journal of Phonetics*.

[21] Diamond SG, Schneider JS, Markham CH (1987). Oral sensorimotor defects in patients with Parkinson's disease. *Advances in Neurology*.

[22] Gräber S, Hertrich I, Daum I, Spieker S, Ackermann H (2002). Speech perception deficits in Parkinson's disease: underestimation of time intervals compromises identification of durational phonetic contrasts. *Brain and Language*.

[23] Ho AK, Bradshaw JL, Iansek R (2000). Volume perception in Parkinsonian speech. *Movement Disorders*.

[24] Hunker CJ, Abbs JH, Barlow SM (1982). The relationship between parkinsonian rigidity and hypokinesia in the orofacial system: a quantitative analysis. *Neurology*.

[25] Kiran S, Larson CR (2001). Effect of duration of pitch-shifted feedback on vocal responses in patients with Parkinson's disease. *Journal of Speech, Language, and Hearing Research*.

[26] Solomon NP, Lorell DM, Robin DA, Rodnitzky RL, Luschei ES (1995). Tongue strength and endurance in mild to moderate Parkinson's disease. *Journal of Medical Speech-Language Pathology*.

[27] Blanchet PG, Snyder GJ (2009). Speech rate deficits in individuals with Parkinson's disease: a review of the literature. *Journal of Medical Speech-Language Pathology*.

[28] Adams SG, Dykstra A, McNeil MR (2009). Hypokinetic dysarthria. *Clinical Management of Sensorimotor Speech Disorders*.

[29] Dromey C (2000). Articulatory kinematics in patients with Parkinson disease using different speech treatment approaches. *Journal of Medical Speech-Language Pathology*.

[30] Goozée JV, Shun AK, Murdoch BE (2011). Effects of increased loudness on tongue movements during speech in nondysarthric speakers with Parkinson's disease. *Journal of Medical Speech-Language Pathology*.

